# Neglect-like characteristics of developmental disregard in children with cerebral palsy revealed by event related potentials

**DOI:** 10.1186/s12883-014-0221-0

**Published:** 2014-11-30

**Authors:** Ingar M Zielinski, Bert Steenbergen, C Marjolein Baas, Pauline BM Aarts, Marijtje LA Jongsma

**Affiliations:** Behavioural Science Institute, Radboud University Nijmegen, PO Box 9104, Nijmegen, 6500, HE The Netherlands; Department of Pediatric Rehabilitation, Sint Maartenskliniek, PO Box 9011, Nijmegen, 6500, GM The Netherlands; School of Psychology, Australian Catholic University, 115 Victoria Pde, Melbourne, VIC 3450 Australia

**Keywords:** Unilateral cerebral palsy, Developmental disregard, Cognitive control, EEG, Event-related potentials, Executive functions, Neglect, Visuo-spatial attention

## Abstract

**Background:**

Children with unilateral Cerebral Palsy (CP) often show diminished awareness of the remaining capacity of their affected upper limb. This phenomenon is known as Developmental Disregard (DD). DD has been explained by operant conditioning. Alternatively, DD can be described as a developmental delay resulting from a lack of use of the affected hand during crucial developmental periods. We hypothesize that this delay is associated with a general delay in executive functions (EF) related to motor behavior, also known as motor EFs.

**Methods:**

Twenty-four children with unilateral CP participated in this cross-sectional study, twelve of them diagnosed with DD. To test motor EFs, a modified go/nogo task was presented in which cues followed by go- or nogo-stimuli appeared at either the left or right side of a screen. Children had to press a button with the hand corresponding to the side of stimulus presentation. Apart from response accuracy, Event-Related Potentials (ERPs) extracted from the ongoing EEG were used to register covert cognitive processes. ERP N1, P2, N2, and P3 components elicited by cue-, go-, and nogo-stimuli were further analyzed to differentiate between different covert cognitive processes.

**Results:**

Children with DD made more errors. With respect to the ERPs, the P3 component to go-stimuli was enhanced in children with DD. This enhancement was related to age, such that younger children with DD showed stronger enhancements. In addition, in DD the N1 component to cue- and go-stimuli was decreased.

**Conclusions:**

The behavioral results show that children with DD experience difficulties when performing the task. The finding of an enhanced P3 component to go-stimuli suggests that these difficulties are due to increased mental effort preceding movement. As age in DD mediated this enhancement, it seems that this increased mental effort is related to a developmental delay. The additional finding of a decreased N1 component in DD furthermore suggests a general diminished visuo-spatial attention. This effect reveals that DD might be a neuropsychological phenomenon similar to post-stroke neglect syndrome that does not resolve during development. These findings suggest that therapies aimed at reducing neglect could be a promising addition to existing therapies for DD.

## Background

Cerebral Palsy (CP) is a group of neurodevelopmental disorders accompanied by disturbances in movement and posture. It is caused by a perinatal non-progressive brain injury and is associated with lifelong motor impairments and disabilities [1,2]. Unilateral CP is among the most common subtypes of CP, comprising 20–40% of the cases [3]. Children with unilateral CP exhibit more pronounced motor deficits on one side of the body, often with the upper extremity more affected than the lower extremity [1,3]. A subgroup of children with unilateral CP also seems to disregard their affected upper limb [4-7]. This so-called Developmental Disregard (DD), leads to a further reduction in using the affected hand in daily life [5,6,8].

Different explanations have been proposed for the phenomenon of DD, all of them giving slightly different indications concerning therapy. Unravelling the underlying factors of DD therefore has high clinical value. Traditionally, DD has been explained by behavioral reinforcement theories [4]. According to these theories, DD can be understood as a result of negative feedback experienced each time the affected hand is used [9,10]. This behavioral phenomenon is akin to the phenomenon of learned non-use that sometimes develops in patients recovering from stroke [11].

More recently, it has been hypothesized that DD could be a phenomenon similar to post-stroke motor neglect syndrome [12]. Similar to children diagnosed with DD, hemiplegic stroke patients with motor neglect show great difficulties in using their affected hand spontaneously, even though strength and coordination are often preserved [13,14]. In line, motor neglect is sometimes confused with learned non-use, leading to misdiagnosis and possible false decisions concerning therapy [14]. However, unlike learned non-use, motor neglect is thought to be the direct result of brain injury to neural networks involved in spatial attention processes [14,15]. The relation between spatial attention and motor deficits has been explained by the premotor theory of Rizzolatti and Carmada [16]. They explain that neural networks for spatial attention are substantially connected with areas that are responsible for motor planning [16]. Brain injury to these networks and resulting deficits of spatial attention therefore leads to an underutilization of the affected body parts related to deficits in motor planning, hence to motor neglect [16].

Apart from the similarities between DD, learned non-use, and motor neglect, a very important factor in children with DD compared to adult stroke patients is the developmental aspect [4]. Studies have increasingly emphasized the important role of developmental factors and the influence of motor learning in understanding DD in children with unilateral CP [4-6,8,17,18]. More specifically, it has been argued that DD results from a lack of use of the affected hand during important developmental periods [5,6]. Due to this lack of use directly related to the initial impaired hand capacity, movements are not being automated and neural substrates serving entire classes of behaviors might not yet be established, refined, or coordinated [6]. This delay in neural refinement does presumably not only affect the actual motor performance of these children, but most likely also the higher order cognitive aspects that are involved in motor behavior [5,17].

Higher order cognitive aspects that are known to be important for motor learning and goal-directed motor behavior and that are strongly determined by developmental trajectories, are executive functions (EFs), also known as cognitive control [19,20]. EF is an umbrella term for different higher order cognitive abilities, such as higher order attentional processes, vigilance, and inhibitory control [21]. It is known that EFs rely on an extensive interconnectivity between different parts of the brain especially involving the prefrontal cortex [22]. As the frontal lobes as well as the intricate connections from underlying brain regions are known to be the last to reach maturity, EFs are known to be the last cognitive area to mature and are therefore especially influenced by developmental periods in middle childhood [22]. Damage to, or delays in development of these white matter tracts are associated with executive dysfunctions [19,21,22].

Within the realm of EFs and goal-directed motor behavior, also known as motor EFs, processes related to attentional control, response switching, as well as response inhibition, are known to be critical to the successful completion of many everyday tasks [19,23]. Furthermore, especially response inhibition has been repeatedly reported to show a progressive development from early childhood and to be frequently impaired in individuals with developmental disabilities [20,24,25]. Based on the assumption that DD can be linked to a delay in the development of motor EFs, we studied whether children with DD experience specific problems in attentional control related to response selection and response preparation as well as in response switching, and response inhibition. This was measured using an adapted go/nogo task. Stimuli within a trial appeared at the left or right side of a laptop screen inducing response switching. Children had to respond correctly to go-stimuli with the hand corresponding to the side of stimulus presentation and inhibit responses following nogo-stimuli.

In order to study motor EFs in children with unilateral CP with and without DD, we measured overt responses in terms of response accuracy. Furthermore, Event-Related Potentials (ERPs) were extracted from the ongoing EEG to register covert cognitive processes involved in this modified go/nogo task. Next to being able to reveal specific neurophysiological correlates of diminished performance, ERPs have the advantage to register covert processes involved in cognitive control even in the absence of overt behavior (e.g. when successfully refraining from responding to nogo- stimuli) [26].

The most commonly identified ERP components within cognitive tasks are the N1, P2, N2, and P3 components [25-27]. Whereas the N1 component has been associated with orienting and early spatial attention processes and the P2 component is known to be modulated by the complexity of the stimuli [26], the later latency N2 and P3 components are known to reflect processes associated with cognitive and attentional control [27]. Accordingly, ERP components that have conventionally been associated with EFs, or cognitive control, during a go/nogo task are the N2 and P3 ERP component [28,29]. The nogo-N2 amplitude is associated with both conflict monitoring and response inhibition and is thought to be generated in the anterior cingulated cortex (ACC) and prefrontal lobe [28,29]. In addition, the P3 amplitude is known to be related to attentional control processes and is thought to be mostly generated in the medial temporal lobe [27,28]. Whereas the nogo-P3 correlates with inhibition control, the go-P3 reflects executive control and is known to be enhanced when demands on cognitive control increase [26,28].

If DD is indeed associated with a developmental delay in motor EFs, these children can be expected to show a diminished performance on the go/nogo task, compared to children with unilateral CP without DD. More specifically, we hypothesized that, (1) children with DD compared to children without DD make more errors when performing the modified go/nogo task reflecting enhanced difficulties in task performance and, that (2) these difficulties would be accompanied by enhanced N2 and P3 ERP components. Next to these group differences, we furthermore expect that (3) developmental changes will be reflected in age differences within the groups.

## Methods

### Participants

Twenty-four children with unilateral CP were recruited from the Sint Maartenskliniek, Nijmegen, the Netherlands. Side of affected hand, manual ability, as well as DD was assessed by an occupational therapist prior to the EEG measurements. Manual ability was assessed using the “Manual Ability Classification System” (MACS), designed to classify how children with CP use their hands when handling objects in daily activities [30]. DD was assessed using the “Video-Observation Aarts and Aarts module: Determine Developmental Disregard” (VOAA-DDD-R) [31]. This structured video observation assesses both the overall duration and frequency of affected upper-limb use. By comparing the affected upper limb use between two standardized tasks, one designed to demand the use of both hands to accomplish the task, whereas the second task is designed merely to stimulate bimanual activity, DD can be determined.

Twelve children were classified as having DD. The other twelve children served as control group (unilateral CP without DD). Even though in each group one child had a visual impairment, they were able to perform the task and showed no differences with respect to response speed or accuracy. They were therefore not excluded from the final analyses. The remaining participants had normal or corrected to normal vision. Furthermore, three of the children were visiting a special school due to an observed delay in general cognitive abilities. Two of them were diagnosed with DD.

To test whether the groups differed with respect to age a Mann-Whitney U-Test was conducted. To test whether there were differences between groups concerning gender, side of the affected hand, and manual ability (MACS), Fisher’s Exact Tests were conducted. For group characteristics and results, see Table 1. Approval for the experiment was obtained from the local Ethical Committee of the Faculty of Social Science (EC) from the Radboud University Nijmegen (Registration number: 2012/049; NL nr: 39607.091.12). The parents of all participants signed a written informed consent.

**Table 1:** **Group characteristics**

	**Unilateral CP**	**Unilateral CP with developmental disregard**	**Statistics**
Age (years; months) (range (years; months))	8; 8 (4; 8–11; 2)	8; 1 (5; 7–12; 11)	Mann-Whitney U Test: **n.s.**
Gender (male/female)	5/7	10/2	Fisher’s Exact Tests: **n.s.**
Affected hand (left/right)	7/5	6/6	Fisher’s Exact Tests: **n.s.**
MACS (range)	1.6 (1–3)	2 (1–3)	Fisher’s Exact Tests: **n.s.**

### Design

**Figure 1:**
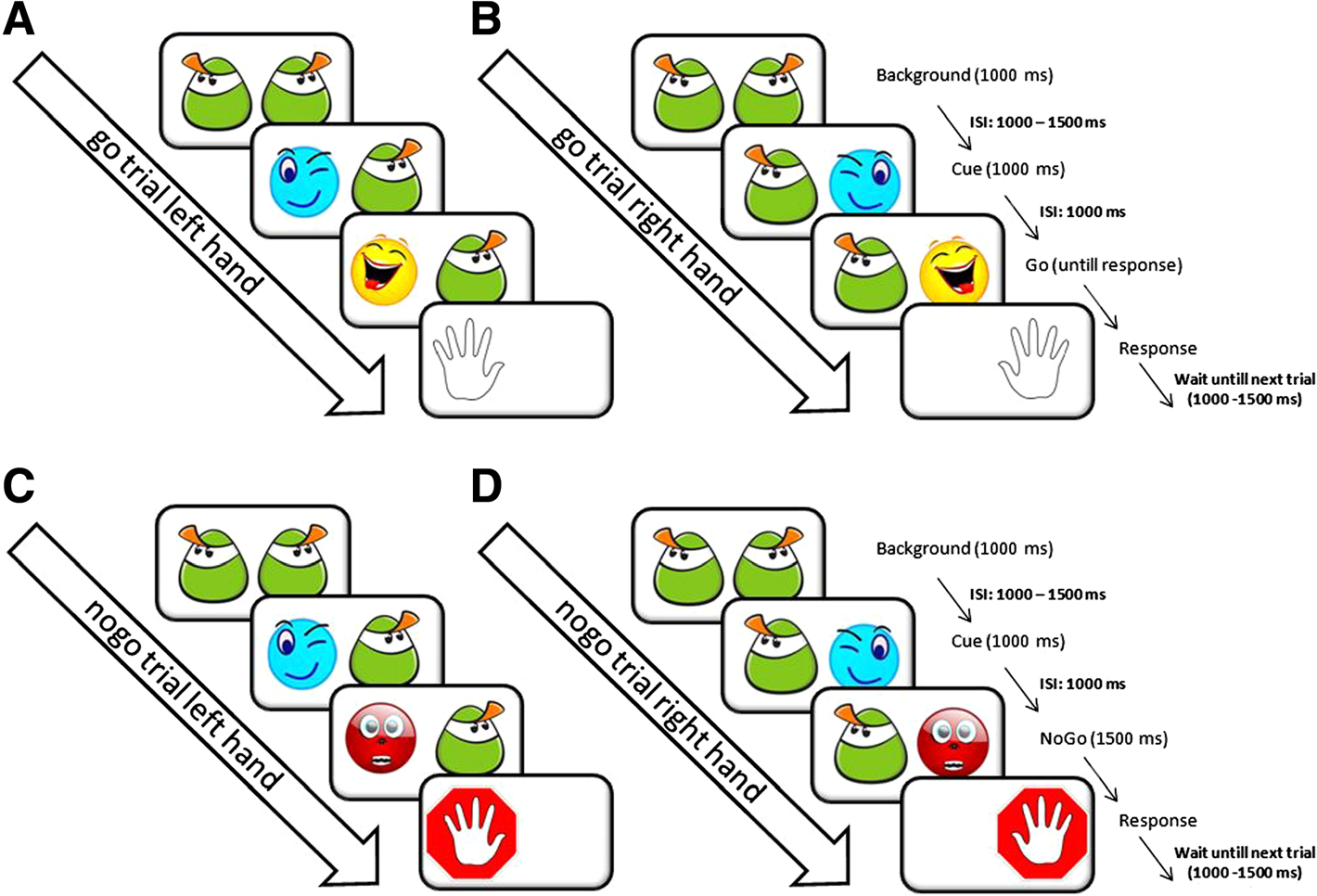
**Trials of go/nogo paradigm.** The four different trials of the modified go/nogo paradigm with a left hand go **(A)** and nogo **(C)** trial and a right hand go **(B)** and nogo **(D)** trial.

In this cross-sectional study a modified go/nogo paradigm [25] was used. Visual stimuli consisting of pairs of “smiley” figures against a white background (size of smileys 7×7 cm) were presented on a laptop screen approximately 40 cm in front of the child. Background-stimuli consisted of two green smileys. Cue-stimuli consisted of a blue paired with a green smiley. Go-stimuli consisted of a yellow smiley paired with a green smiley. Nogo-stimuli consisted of a red smiley paired with a green smiley. Go- and nogo-stimuli were always presented at the same side as the preceding cue-stimulus. Figure 1 provides a graphical presentation of the stimuli. Trials consisted of one to three background-stimuli and a cue-stimulus, followed by either a go- or nogo-stimulus.

Thus, four different types of trials were presented: go-trials for the non-affected hand, go-trials for the affected hand, nogo-trials for the non-affected hand and nogo-trials for the affected hand. Each trial type was presented 20 times. Trials (n = 80) were presented in a random order, demanding regular response switching and response inhibition with respect to both hands. The stimulus duration was 1000 ms for background- and cue-stimuli, 1500 ms for nogo-stimuli, and for go-stimuli until a response was made. The inter-stimulus interval (ISI) between cue- and go/nogo-stimuli was kept fixed at 1000 ms. Participants were instructed to respond as quickly as possible to go-stimuli. For this purpose two red buttons (diameter: 9.5 cm; height: 5.5 cm) were located next to the laptop keyboard, one at the right side and one at the left side. The distance between these buttons was kept at 30 cm to prevent that the wrong hand was used to press the according button. After each correct response to a go-stimulus a short laughing sound was presented. After each correct inhibited response to a nogo-stimulus a short trumpeting sound was presented. Errors were defined as false hits following cue- and nogo-stimuli as well as omissions following go-stimuli (no response within 2000 ms).

### Electrophysiological recordings

EEG signals were recorded with a 32-channel active electrode system (actiCap MedCaT B.V. Netherlands) and amplified by a 32-channel BrainAmp EEG amplifier with electrode placement according to the international 10–20 system at Fz, FCz, Cz, Pz, Oz, Fp1/2, F3/4/7/8, FC1/2/5/6, C3/4, T7/8, CP1/2/5/6, P3/4/7/8, O1/2 [32,33]. A ground electrode was placed over AFz. The EEG signal was offline re-referenced to linked mastoids and stored on disk for offline analyses. Electrooculography **(**EOG) was recorded with bipolar channels placed above and below the right eye and on the outer canthi of each eye. Electrode impedance was kept below 5 kΩ. The signal was digitized at 1000 Hz between 0.016–250 Hz. For each participant an ocular correction was applied [34] and segments containing artifacts exceeding ±150 μV were removed. The EEG signal was detrended and off-line filtered between 1–24 Hz. Next, the EEG was segmented from -250 ms to 750 ms related to stimuli and baseline corrected (-250 - 0 ms). Epochs corresponding to incorrect trials were excluded (total of 7.9% of the trials). Segments were averaged per stimulus type (cue vs. go vs. nogo) and hand (affected vs. non-affected).

### Data analysis

Errors and Reaction Times (RTs) of all correct responses were analyzed using repeated measures general linear model (GLM) analysis with handedness (affected vs. non-affected hand) as independent within-subject variables and group (control group vs. DD group) as between-subject factor.

The ERP N1, P2, N2, and P3 component amplitudes at Fz, FCz, and Cz were further analyzed. To allow blind scoring, ERP amplitudes were defined as the averaged value within a fixed latency window: N1 (120–140 ms), P2 (220–240 ms), N2 (320–360 ms), and P3 (500–600 ms). ERP components were analyzed using repeated measures GLM analyses with handedness (affected vs. non-affected hand) and electrode side (Fz, FCz, and Cz) as independent within-subject variables and group (control group vs. DD group) as between-subject factor.

Whenever interaction effects were observed, appropriate T-Tests were performed. To account for multiple testing, a Bonferroni correction was applied. Whenever group differences were observed, multivariate linear regression analyses were applied with age as independent variable. This was done to explore possible developmental changes within the groups that might explain the group differences. For all analyses, the significance level was set at α < .05.

## Results

**Figure 2:**
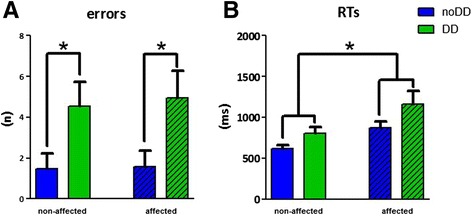
**Errors (A), and RTs (B).** Depicted are means ± SEMs. Blue bars show the results of the CP children without DD (noDD); green bars show the results of children with DD (DD). Clear bars depict the results of the non-affected hand; striped bars depict the results of the affected hand.

With respect to the behavioral data, the analyses of errors revealed a significant group effect (F(1,22) = 22.83; *p* = .023; η_p_^2^ = .213; 95% CI, .48 to 5.94). Children with DD made significantly more errors. No interactions were found (see Figure 2A). The multivariate linear regression analysis with age as independent variable and total errors for both hands as dependent variables did not reveal any predictable value of age for the amount of mistakes for either group.

In addition, the RT data revealed a significant hand effect (F(1,22) = 11.24; *p* = .003; η_p_^2^ = .338; 95% CI, 37.91 to 160.93), showing that children across both groups responded significantly slower with their affected hand. No group effect with respect to the RTs was found (see Figure 2B).

**Figure 3:**
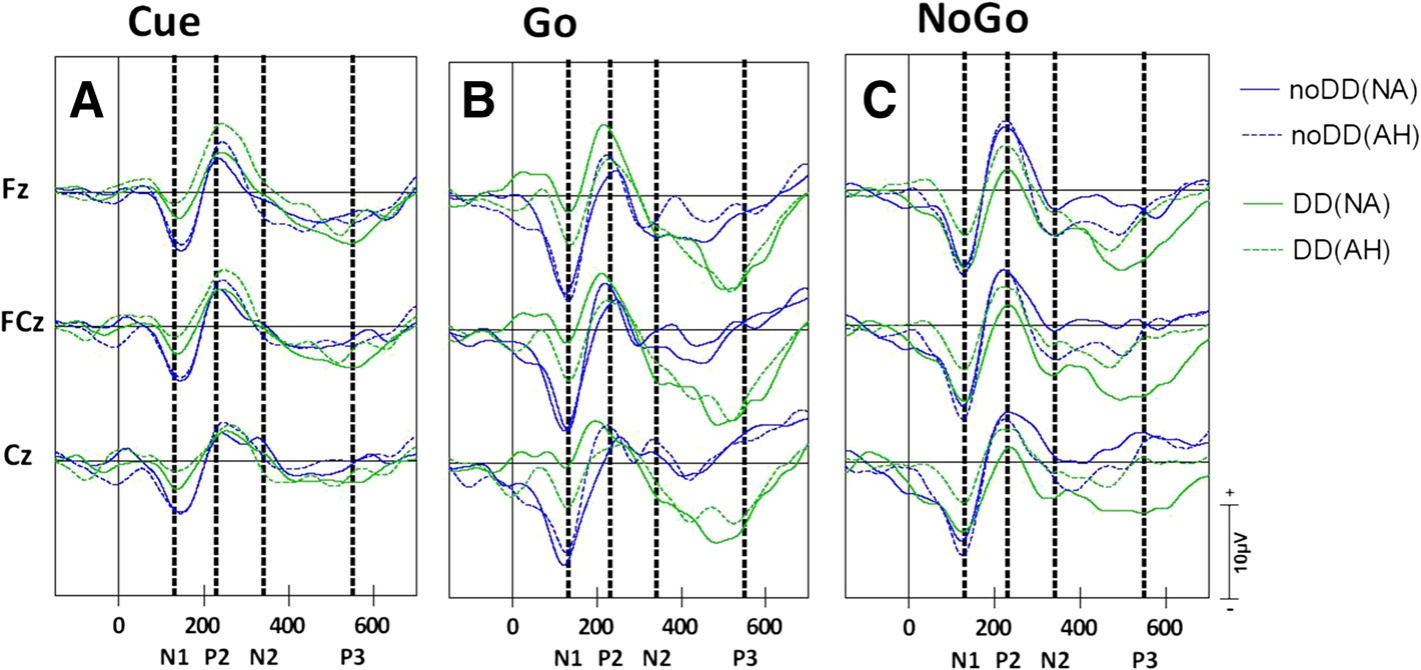
**Grand averaged waveforms.** Grand average ERPs following cue- **(A)**, go- **(B)**, and nogo-stimuli **(C)**. ERPs of children with unilateral CP without DD (noDD) are depicted in blue and for children with DD (DD) in green. ERPs for the non-affected hand are depicted in solid lines (NA) and for the affected hand in dotted lines (AH).

The grand average ERPs following cue-, go-, and nogo-stimuli contained a clear N1 (mean latency: 130 ms), P2 (mean latency: 230 ms), and N2 (mean latency: 340 ms) component. Instead of a classic P3, a late latency negative wave was observed following go- and nogo-stimuli (mean latency: 550 ms). This fronto-central negative wave in children has earlier been reported to be comparable to the classic P3 wave in adults [35]. Grand average ERPs at Fz, FCz, and Cz electrode location following cue-, go-, and nogo-stimuli for both the DD and control group (CP without DD) are depicted in Figure 3.

**Figure 4:**
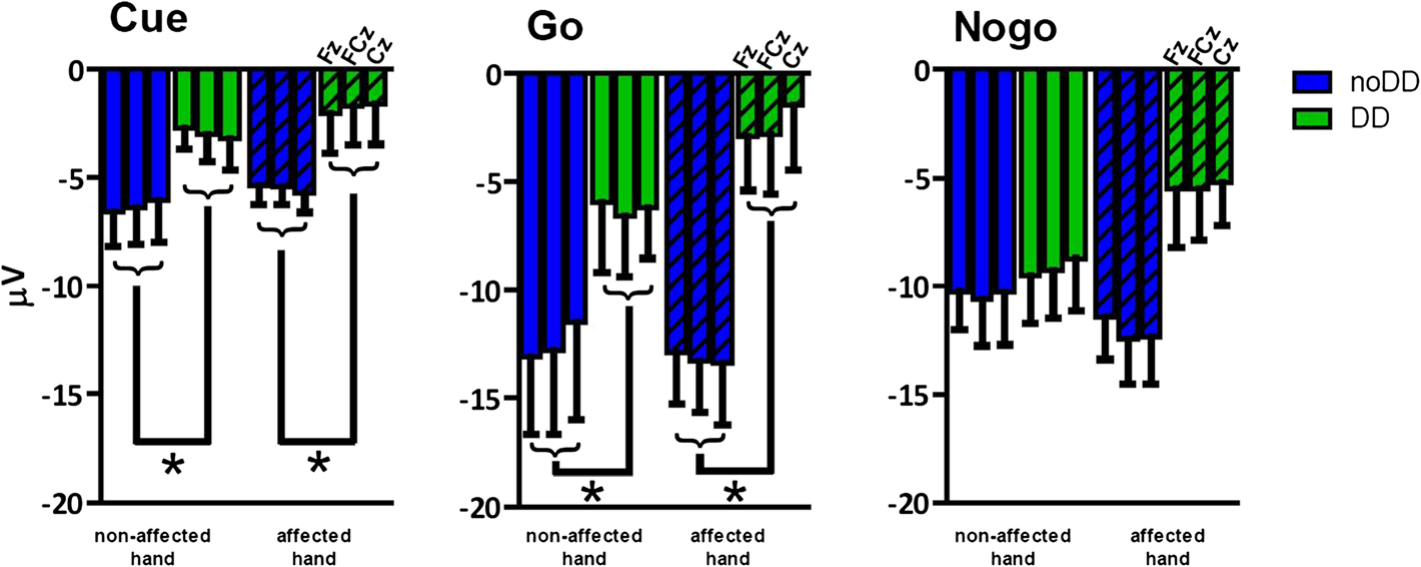
**N1 amplitudes (mean ± SEM; Fz, FCz, Cz) to cue-, go-, and nogo-stimuli.** Children without DD are depicted in blue bars (noDD) and children with DD in green bars (DD). Clear bars depict the results of the non-affected hand; striped bars depict the results of the affected hand.

The analyses of the N1 ERP component revealed a main effect of group following cue- (F(1,22) = 7.01; *p* = .015; η_p_^2^ = .242; 95% CI, 0.77 to 6.36) and go-stimuli (F(1,22) = 5.36; *p* = .030; η_p_^2^ = .196; 95% CI, 0.89 to 16.10). The N1 component was diminished in the DD group compared to the control group (Figure 4). In addition, a hand∗group interaction was found following nogo-stimuli (F(1,22) = 4.78; *p* = .04; η_p_^2^ = .178). However, post-hoc analyses (Independent-Samples T-Test) revealed no significant group or hand effects. Furthermore, for both the cue- and go-stimuli, the multivariate linear regression analyses per group, with age as independent variable, and N1 amplitude at different electrode positions as dependent variables, did not reveal any age effects.

With respect to the P2 component only electrode effects were found following cue- (F(2,21) = 6.58; *p* = .006; η_p_^2^ = .385) and go-stimuli (F(2,21) = 6.29; *p* = .007; η_p_^2^ = .375). Also, following cue-stimuli a hand∗electrode interaction was found (F(2,21) = 3.93; *p* = .036; η_p_^2^ = .272). Post-hoc analyses of this interaction (Paired Samples T-Test) revealed no hand effect at the separate electrode sides.

The analyses of the N2 component also revealed significant electrode effects following go- (F(2,21) = 6.14; *p* = .008; η_p_^2^ = .369) and nogo-stimuli (F(2,21) = 5.54; *p* = .012; η_p_^2^ = .345). In addition, an electrode∗group interaction was found following go-stimuli (F(2,21) = 4.25; *p* = .028; η_p_^2^ = .288). Post-hoc analyses (Independent-Samples T-Test) revealed no differences between groups at the different electrode sides.

**Figure 5:**
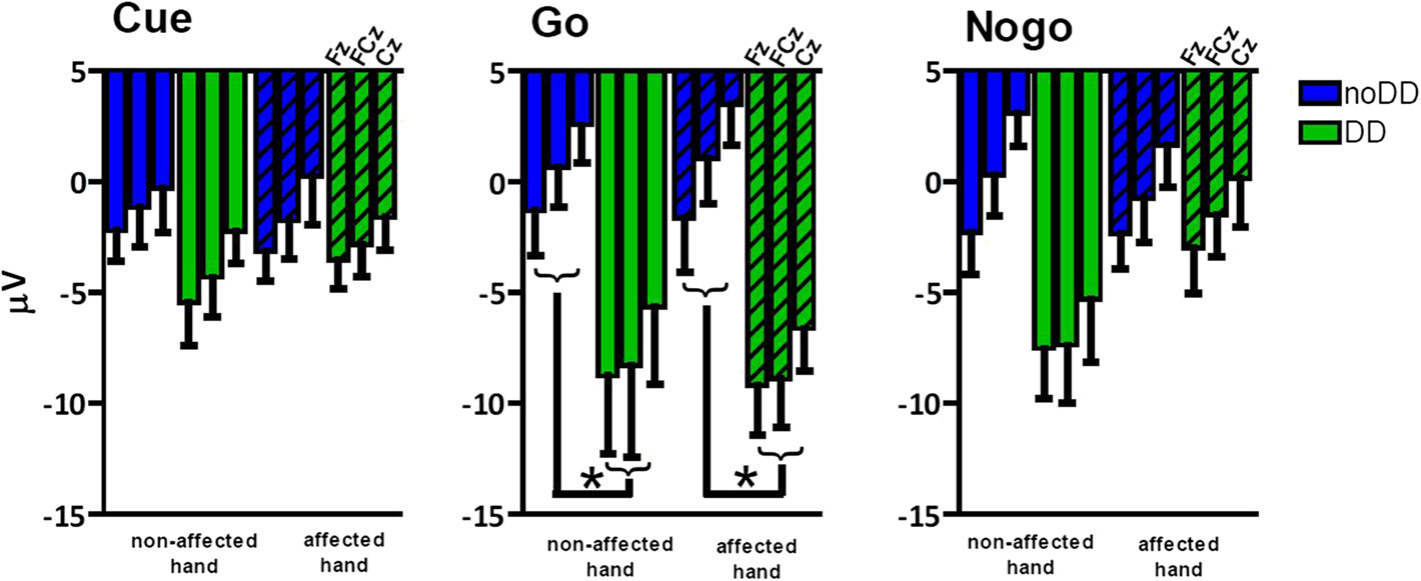
**P3 amplitudes (mean ± SEM; Fz, FCz, Cz) to cue-, go-, and nogo-stimuli.** Children without DD are depicted in blue bars and children with DD in green bars. Clear bars depict the results of the non-affected hand; striped bars depict the results of the affected hand.

Next to significant electrode effects following cue- (F(2,21) = 10.55; *p* = .001; η_p_^2^ = .501), go- (F(2,21) = 8.29; *p* = .002; η_p_^2^ = .441), and nogo-stimuli (F(2,21) = 8.97; *p* = .002; η_p_^2^ = .452), the analyses of this P3-like ERP component revealed a main effect of group following go-stimuli only (F(1,22) = 9.00; *p* = .007; η_p_^2^ = .290; 95% CI, - 14.72 to - 2.69). Following go-stimuli, the P3 ERP component was enhanced in the DD group compared to the control group (CP without DD). No interactions were observed for either stimulus (see Figure 5).

**Figure 6:**
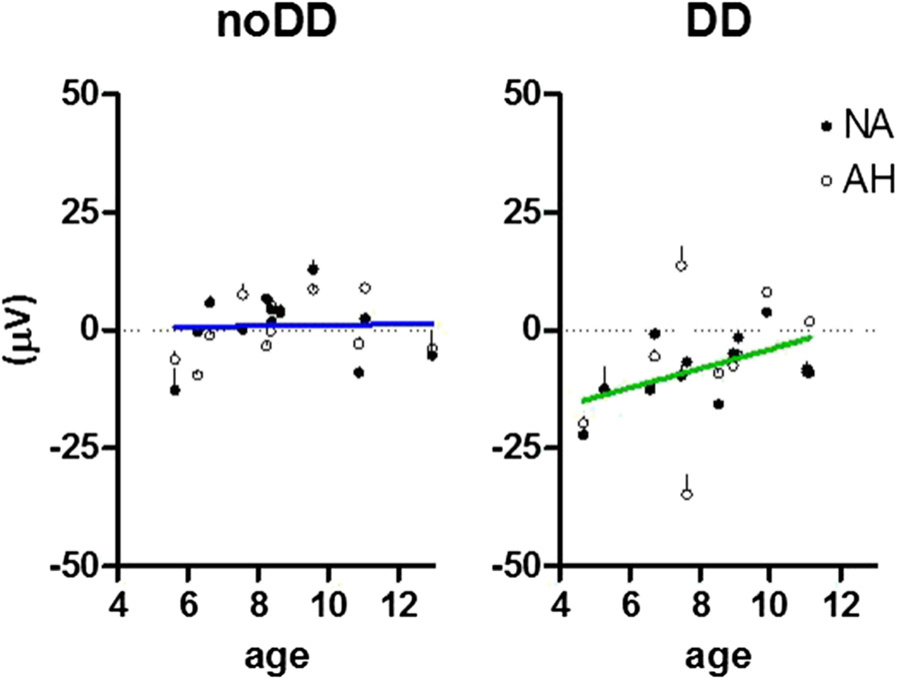
**Mean P3 amplitudes with age (mean ± SEM of Fz, FCz, Cz per participant) to go-stimuli.** Children without DD are depicted on the left (noDD) and children with DD on the right (DD). Data of the non-affected hand (NA) are depicted as dots and for the affected hand (AH) as circles. The regression line for children without DD is depicted in blue and for children with DD in green.

The multivariate linear regression analysis with age as independent variable and P3 amplitude at different electrode positions as dependent variables did reveal significant predictable value of age for the amplitude of the go-P3 component in the DD group (*R*^*2*^ = .402; *p* = .027). The older the children in the DD group, the less enhanced the P3 component was. This was not the case in children without DD (see Figure 6).

## Discussion

The goal of the current study was to test the hypothesis that children with Developmental Disregard (DD) experience deficits that are directly related to a developmental delay in executive functions (EFs) involved in goal-directed motor behavior, also known as motor EFs. Under the assumption that DD might be associated with a delay in motor-skill development [5,6] as well as a broader delay in the development of motor EFs [5,17], we expected that children with DD would demonstrate impaired performance during a modified go/nogo task. Next to evaluating the overt responses measured in terms of response accuracy, covert cognitive processes involved in attentional control related to response selection and response preparation as well as in response switching, and response inhibition were registered using Event-Related Potentials (ERPs) extracted from the ongoing EEG. We expected children with DD to (1) make more errors and to (2) show enhanced late latency N2 and P3 ERP components as these components have been reported to reflect covert cognitive control processes when elicited in a go/nogo task [26,28]. We furthermore expected that (3) if DD is related to a developmental delay in motor EFs, these effects would diminish with age.

With respect to the first hypothesis, our behavioral results indeed showed that children with DD, compared to children without DD, made more errors during the modified go/nogo-task. Therefore, our first hypothesis was confirmed. This increase was however not restricted to an increase in false hits (i.e. diminished response inhibition), or misses (i.e. diminished response switching), but rather reflected in the total amount of errors. Thus, children with DD do not seem to experience any specific difficulties related to response inhibition or response switching, but seem to experience general difficulties when completing the modified go/nogo-task. These general difficulties did however not seem to be determined by developmental aspects, as age in neither group did predict the number of errors. With respect to our behavioral findings, our third hypothesis was therefore not confirmed.

To examine the underlying cognitive factors that might have contributed to this increase of errors, the ERPs following the different stimuli were inspected. Our ERP data of the late latency N2 and P3 components showed a similar pattern of results as the behavioral data. Our second hypothesis was partly confirmed: Although no group differences with respect to the N2 and P3 components following nogo-stimuli were observed, children with DD showed an enhanced fronto-central P3 component following go-stimuli. Nogo-N2 and nogo-P3 amplitudes are respectively correlated with response inhibition and inhibition control processes [28,29]. As there were no differences between groups regarding these components, it seems that children with DD do not experience any specific difficulties in response inhibition processes compared to children with unilateral CP without DD. This is in line with the behavioral findings, as the increased errors were not restricted to false hits following nogo-stimuli.

The go-P3 component, which was found to be enhanced in the DD group, is known to reflect executive control processes preceding the motor response [28]. In this respect it has often been reported that the go-P3 is affected by the mental effort that the participant devotes to the task, so that increased mental effort is accompanied by an increase in the P3 amplitude [17,26,28]. As such, the current finding of an enhanced P3 following go-stimuli in children with DD might reflect increased mental effort serving executive or cognitive control mechanisms involved in goal directed behavior. With respect to the go-P3, it was furthermore found that only in the DD group, age had a predictable value on the P3 amplitude. With respect to the go-P3 component our third hypothesis was therefore confirmed. As children with DD developed, the enhancement of the go-P3 amplitude was reduced. It might therefore be concluded that the differences between the two groups might be best explained by a developmental delay of executive control mechanisms involved in goal directed motor behavior in children with DD.

In all, our findings add to the accumulating evidence that the performance difficulties observed in children with DD might be related to a disproportional amount of attentional control needed during motor performance [5,17]. Indeed, in our previous ERP study we also observed that children with DD allocated more mental effort when preparing a response with the affected hand in a dual-hand cued-target paradigm [17]. Furthermore, the finding that for children with DD age is significantly related to the amount of cognitive control involved in response preparation is in line with the assumption that developmental factors play an important role in the development and persistence of DD [4-6,8,17,18]. However, as the total amount of errors in our current study was not related to age in either group, there seemed to be additional cognitive factors, next to the developmental related cognitive control processes, contributing to the observed performance deficits in the DD group. One possible explanation was given by the additional ERP findings of the mid-latency N1 component of the current study.

Next to the differences between groups in the total amount of errors and the late-latency go-P3 component, the electrophysiological results of the current study revealed distinctive effects on the mid-latency N1 ERP component. Following both cue- and go-stimuli, the N1 component was decreased in children with DD compared to children without DD. However, there was no relation with age observed in either group related to the N1 amplitude.

The N1 ERP component, a negative going wave occurring at approximately 120 ms after presentation of a visual stimulus, is known to be particularly modulated by early orienting and spatial attention processes [36-40]. The visual N1 component in particular, reflecting the activity within the intraparietal sulcus (IPS) in the dorsal parieto occipital cortex, has been stated to reflect bottom-up stimulus processing at a level dealing with spatial attention and visuomotor control [27,41]. Interestingly, the N1 component following cue- and target-stimuli has already been found to be diminished in patients with a post-stroke neglect syndrome [41,42]. From the current finding of a diminished N1 component in children with DD, it may therefore be concluded that these children experience difficulties in stimulus processing at a level dealing with spatial attention and visuomotor control similar to that observed in post-stroke neglect. In line with this conclusion, Sutcliffe, Logan and Fehlings [12] indeed stated that the clinical symptoms of DD can be conceived of as a phenomenon similar to post-stroke neglect syndrome leading to an underuse of one side of the body unrelated to the impaired movement capacity [15,43].

As stated above, there was no relation observed between age and the N1 amplitude. This finding suggests that development does not influence these deficits in spatial attention and visuomotor control in children with DD. This might be explained by the premotor theory of Rizzolatti and Carmada [16]. In this theory it is stated that motor deficits observed in motor neglect patients can be explained by an underlying injury to neural networks of spatial attention as these neural networks are substantially connected with areas that are responsible for motor planning and preparation [16]. This theory, in combination with our current findings, leads to the suggestion that in children with DD neural networks involved in spatial attention seem to be affected. This would mean that in children with DD, next to their developmental delay in cognitive control processes related to motor behavior, different neural networks seemed to be affected than in children with unilateral CP but without DD. In future studies, this question could be addressed using neuroimaging techniques that are able to look at these specific neural networks.

Interestingly, in the current study all observed effects (enhancement of errors; enhanced go-P3; decreased cue- and go-N1) were not restricted to the affected side. This finding might be explained by the neurocognitive perspective on DD proposed by Houwink and colleagues (2011) [5]. They proposed that DD can be explained by the high attentional demands associated with the use of the affected upper limb [5]. The attentional demands of the go/nogo-task used in the current study were higher than in a simple cued-target paradigm, as has been used in earlier studies [17,41]. It might therefore be concluded that this enhancement of attentional demands led to a general decrease in stimulus processing and response preparation within the DD group.

Study limitations of the current study include the heterogeneity of the studied group concerning their etiology as well as the underlying differences in brain injury. This limitation is however inherent to the participant population, as unilateral CP comprises a very heterogeneous group of movement disorders [1,3]. Further limitations of the current study relate to the limited data on the cognitive and perceptual information of the individual child. For future studies it should be considered to add a short screening procedure to gain some quantitative information on the cognitive functioning as well as the perceptual processing.

## Conclusions

The current study shows that next to difficulties in motor executive functions (EFs) that diminish with age, children with DD show neglect like symptoms that do not seem to resolve during development. This implies that therapies aimed at reducing motor neglect could be a promising addition to existing therapies for DD. Rather than only counter-conditioning learned non-use, as in Constraint Induced Movement Therapy (CIMT) [44,45], or automating dual hand performance, as in bimanual training therapies (BiT) [46], therapies aiming at reducing DD should also account for possible spatial attention deficits. To our knowledge, there are no therapies for children with unilateral CP directly aiming at reducing motor neglect. Future research should therefore be directed at studying the efficacy of therapies that are based on training children with DD to attend voluntary to their contralesional space. This is for example done in limb activation training (LAT) that is already applied to adult neglect patients [47].

## Abbreviations

BiT: Bimanual training
CIMT: Constraint induced movement therapy
CP: Cerebral palsy
CVA: Cerebrovasculair accident
DD: Developmental disregard
EEG: Electroencephalography
EOG: Electrooculography
ERP: Event-related potential
GLM: General linear model
ISI: Inter stimulus interval
LAT: Limb activation training
